# Ceftriaxone-induced hepatotoxicity in patients with common medical infections in Qatar: A retrospective study

**DOI:** 10.5339/qmj.2022.27

**Published:** 2022-07-07

**Authors:** Manish Barman, Bassem Al Hariri, Abdul Rahman Mustafa, Naseem Ambra, Israa Amjed, Ahmad Eid Nazzal Alharafsheh, M.N. Illahi, S. Hamuda, Mohamedali Gaafar, Muhammad Sharif

**Affiliations:** ^1^Department of Medicine, Hamad Medical Corporation, Doha, Qatar E-mail: drbarman@yahoo.com; ^2^Department of Clinical Medicine, Weill Cornell Medicine, Qatar

**Keywords:** Drug-induced liver injury, drug-induced hepatitis, drug-induced cholestasis, Ceftriaxone

## Abstract

Introduction: Ceftriaxone, a third-generation cephalosporin, is frequently used for the treatment of various bacterial infections as a broad-spectrum antibiotic for many decades. Although ceftriaxone is a well-tolerated drug in most cases, it can lead to serious liver injury, which can be a real challenge to the treating physician. Given the potentially serious adverse effects that can vary from mild biochemical abnormalities to complete liver failure, we intend to assess the spectrum of liver injury based on biochemical criteria for patients treated with ceftriaxone for common bacterial infections in Qatar.Objectives: This study aimed to explore the incidence of ceftriaxone-induced liver injury at Hazm Mebaireek General Hospital, Qatar, and to evaluate the relationship of the ceftriaxone dose, if any, with liver dysfunction.Methods: This retrospective study included hospitalized adult patients treated with ceftriaxone at our hospital from January 2019 to December 2019 and analyzed demographic and clinical data obtained from electronic medical records. This study determined the incidence of liver injury (primary outcome) in patients treated with ceftriaxone (2 g/day) for ≥ 2 consecutive days by reviewing liver function test results until the day of discharge and at the first outpatient follow-up.

Results: The final data analysis included a total of 634 patients admitted and treated with ceftriaxone from January 2019 to December 2019.In the multivariate analysis with propensity score adjustment, ceftriaxone was independently associated with liver injury, especially when combined with other agents utilizing hepatic metabolism.Conclusions: Ceftriaxone was associated with a significantly higher incidence of liver injury (19.7%) when used along with other medications that are metabolized in the liver, as found in the present study compared with other similar studies (approximately 2.9%–13.9%). Furthermore, the incidence was too high to be ignored in clinical practice.

## Introduction

Multiple drugs undergo hepatic metabolism, and drug-induced liver injury (DILI) is a significant medical concern that remains a challenge to the treating physician and clinical pharmacists. Fortunately, most DILI cases are benign, and patients mostly improve after the offending drug is discontinued timely to prevent worsening or permanently causing liver damage. According to various international DILI registries, amoxicillin–clavulanate remains one of the drugs most frequently associated with hepatocellular toxicity along with antituberculosis drugs, such as isoniazid and rifampin.^1–4^ Ceftriaxone is a broad-spectrum, third generation long-acting cephalosporin. It has been in clinical use for more than three decades and remains one of the most preferred drugs against infectious diseases.^5–8^ It reversibly binds to plasma proteins and has an elimination half-life of 6–9 h. It is mainly eliminated via urine (67%) and partially through feces via bile secretions.^9^

Consistent observations have been reported in many patients developing hepatocellular dysfunction when treated with antibiotics, often not explainable to other causes. Liver injury may progress from mild biochemical anomalies to severe hepatitis. Till date, the exact mechanism of injury is unclear and presumed most likely to be an idiosyncratic reaction that might involve metabolic or immunological pathways^5,6,10^ causing direct hepatocyte toxicity and even leading to apoptosis and some elements of cell necrosis in some cases. One issue regarding ceftriaxone is related to its proclivity toward calcium precipitation, which leads to the formation of insoluble crystals in bile secretions precipitating biliary sludge and resulting in gall bladder calculi.^11^ Many case reports^12–20^ and prospective studies^21–29^ have cited biliary pseudolithiasis in both adults and young children. Shiffman et al.^30^ also suggested that dosages of ceftriaxone ( ≥ 2 g daily) and conditions that impair gallbladder contractility may act as predisposing factors. However, biliary precipitation is self-limiting, and sludge mostly resolves after ceftriaxone discontinuation.^31^ Weaver et al.^32^ elucidated various other postulated mechanisms of liver injury, including mitochondrial and lysosomal impairment, reactive metabolites, and immune-mediated injury. The different types of DILI and their prognosis are presented in Table 1.

In our clinical practice too, like others, we have encountered many patients with liver dysfunction following ceftriaxone use. Therefore, the researchers were inclined to explore causal effects, if any, and to determine whether this observation is a legitimate cause of concern. To the best of our knowledge, no detailed reports are available regarding the incidence of liver injury among adult patients treated with ceftriaxone in Qatar. This study aimed to evaluate the association of liver injury with ceftriaxone use when treating common bacterial infections and to determine other variables associated with liver injury among adult patients from a wide range of backgrounds and diverse genetic pool, as Qatar is home to people from all over the world. This study looks at the occurrence of ceftriaxone-induced liver injury presented as elevated levels of transaminases in current clinical practice and explores any plausible predisposing factors among our patients.

## Methods

### Study design and population

This retrospective cohort study analyzed the association of ceftriaxone use with the incidence of DILI in all patients admitted at Hazm Mebaireek General Hospital for common medical conditions that required ceftriaxone antibiotic therapy, as clinically indicated. The inclusion criteria were as follows: a) hospitalized patients aged ≥ 18 years, from January 2019 to December 2019, at our hospital and b) patients who were administered ceftriaxone intravenously ≥ 2 g/day for ≥ 2 consecutive days. The exclusion criteria were as follows: a) patients aged of < 18 years, b) changing the doses of ceftriaxone during the treatment period, c) re-administration of ceftriaxone within 2 weeks after the discontinuation of ceftriaxone, d) patients who did not undergo assessments of serum biochemistry more than once after the administration of ceftriaxone, and e) any underlying liver diseases or malignancies.

### Clinical data collection

Clinical data for the eligible patients were collected by reviewing their inpatient electronic medical records, including demographic characteristics, comorbidities, duration of ceftriaxone treatment, site of infection, concomitant drugs used, and laboratory data of both liver and renal functions. Aspartate aminotransferase (AST), alanine aminotransferase (ALT), alkaline phosphatase (ALP), and total bilirubin (activity) levels, which are markers of adequate liver function, were evaluated separately. The serum biochemistry information was evaluated during treatment with ceftriaxone and at discharge.

### Outcomes, statistical consideration, and data analysis

In this study, the primary outcome was a liver injury that occurred >48 h after ceftriaxone treatment. By definition, DILI^8^ requires reasonable elimination of common etiologies that affect the liver when the patients have taken any medication and may include one of these parameters: (1) ≥ 2-fold elevation above the upper normal limit of ALT (ULN = 55 IU/L) and (2) a combined increase in AST, ALP, and total bilirubin (activity) levels provided that one of them shows >2-fold elevation above the ULN. Laboratory reference values considered were as follows: ALT (ULN = 55 IU/L), AST (ULN = 40 IU/L), ALP (ULN = 120 IU/L), and total bilirubin (TB) ≤ 2.4 mg/dL.

The American College of Gastroenterology guidelines^8^ suggest that the R-value obtained via observations in ALT and ALP activities can reflect the pattern of liver injury. However, the researchers have not ventured into this detail, as it is out of scope for the current analytical review. The incidence rate was determined by including the number of patients who manifested signs of liver injury through deranged liver function tests and dividing that by the total study population at risk included in our study. The secondary outcomes included causal associations, predisposing factors such as age group, comorbidities, associated medication use, smoking, and alcohol use for liver function test deterioration, if any.

### Statistical methods

The multivariate analysis for age, body mass index (BMI), concomitant drugs, comorbidities, site of infection, alcohol, and smoking habits was undertaken, and the Chi-squared test was used to evaluate the presence of significant difference (*p* < 0.01) between the derived results and the expected outcomes. Categorical variables are presented as numbers and percentages, which were analyzed using the χ2 test and Fisher's exact test with a 95% confidence interval (CI). The incident rate and 95% CI values were estimated from the population size, and differences between the groups were considered significant at *p* < 0.01

## Results

### Study population selection based on characteristics and antibiogram

**Figure 1.**
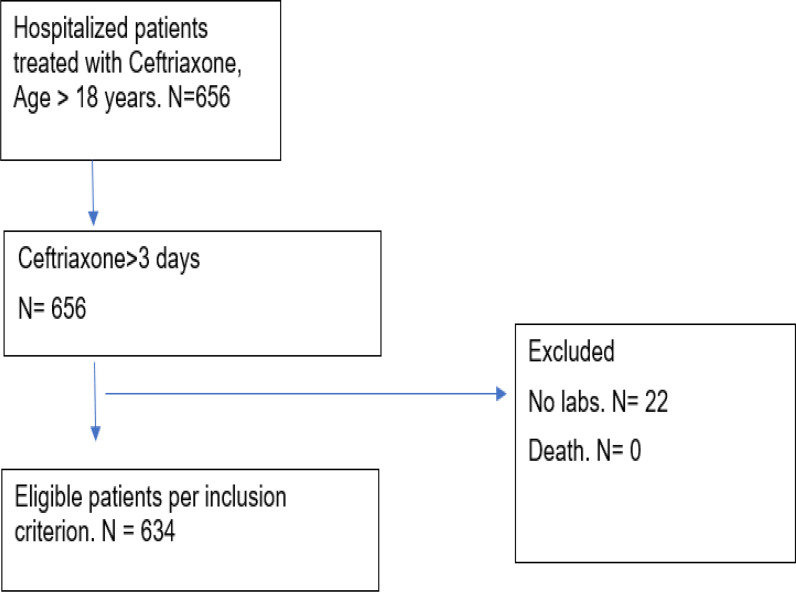
Flow chart of patient selection

From January 2019 to December 2019, a total of 656 patients aged ≥ 18 years received ceftriaxone treatment (2 g intravenous/q24 h) for ≥ 3 consecutive days. A total of 22 patients were excluded because they did not undergo an assessment of serum biochemistry more than once during treatment and 2 weeks after the discontinuation of ceftriaxone (n = 22). Thus, only 634 patients were included in the analysis (Figure 1). A high dose of ceftriaxone (>2 g intravenous /q24 h) was administered to only 14 (2.2%) patients, whereas the remaining 620 (97.8%) patients received a normal dose ( < 2 g intravenous/q24 h). Renal parameters were verified to validate that all study patients had estimated glomerular filtration rate > 10 not requiring any ceftriaxone dose modifications. Baseline population characteristics are presented in Table 2. The high-dose group could not be analyzed separately in view of the very limited number of patients.

### Antibiogram of the study population

**Figure 2.**
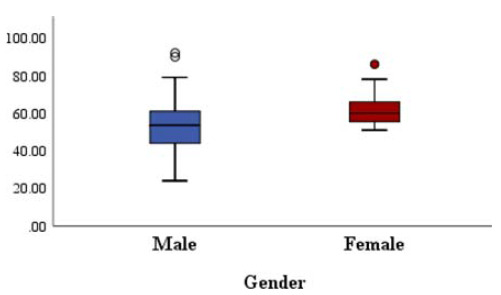
Bacterial culture growth isolates in the patient population.

We further collected data of the bacterial culture growth isolated in our study population, as depicted in Figure 2. The total culture isolates percentage is more than hundreds due to some mixed culture growths in a few samples and because some patients had more than one culture samples documented. Table 3 shows the empiric antibiotics of choice for common clinical infections used in our hospital.

The primary outcome defined as an abnormal liver function test results >48 h after intravenous administration of ceftriaxone gave us an incidence rate {number of patients reporting liver dysfunction (125) / total number of patients where ceftriaxone was used (634)} = 19.71%. For the secondary outcomes, the multivariate analysis by age, BMI, comorbidities such as diabetes mellitus and hypertension, or infection site did not reveal any significant differences (*p*>0.01). However, significant observations of associations were noted in the group with concomitant drugs, mainly paracetamol and azithromycin. Alcohol use was associated with some significance (*p* < 0.05), but smoking was certainly not.

## Discussion

No published reports have focused on the incidence of liver injury in the adult population treated with ceftriaxone in Qatar and the association between comorbidities and lifestyle factors. Antimicrobial-related adverse events including hepatotoxicity are reported widely in the medical literature. Most cases are idiosyncratic in nature, as they cannot be predicted from the drug pharmacological profile or preclinical tests. It may be an immunological response or related to other concomitant factors including hepatotoxic metabolites. However, it is difficult to single out specific medication, as treatment consistently involves multiple associated parameters. Given the inherent challenges in recognizing and reporting DILI, the incidence is difficult to determine. The annual incidence of DILI that has been reported across European studies ranged from 2.3 to 13.9 in various population-based studies.^9,33–34^

Usually, patients with high-risk status are those who have previous adverse reactions to antibiotics, have multiple comorbidities, or have baseline impaired hepatic function in the absence of close monitoring. Thus, it is pertinent to carefully balance potential risks with expected benefits in secondary care. Precision medicine utilizing the new genome-wide affiliation holds the potential for better understanding and outcomes in near future. However, until then, it is best to be aware of the clinical implications of commonly used antibiotics. Regulatory bodies have tried to raise awareness in recent years by targeting specific antibiotic usage, helping physicians at large to better identify and minimize the potential harm related to adverse reactions.^35–37^ However, multiple factors complicate patient care. First, patients’ factors such as age, genetic predisposition, comorbidities, concurrent medications, smoking, and excessive alcohol consumption may all increase vulnerability to drug-induced hepatotoxicity^38^ although their exact mechanisms remain uncertain, with paracetamol as the most commonly used along with antibiotics particularly those metabolized through the hepatic channel.

Second, hepatotoxicity appears to be often related to the administration of large doses of any drugs, with 77% of DILI cases included in the Swedish registry occurred with any drugs administered.^17,38^ Third, initial liver injury, as detected by an increase in transaminases, may be transient despite continued treatment, unless the patient has additional factors that will cause mild toxicity to turn into severe hepatic dysfunction.^29^ Fourth, antibiotic-associated complete hepatic failure is still rare (one case per one million adults/year).^36^ Finally, the retrospective analysis of DILI remains a menace because of the inherently subjective nature, complexity of the disease, and potential observer bias, creating confusion among healthcare professionals since the onset of liver dysfunction is often widely variable.^16,25,35,36^ Most patients often recover after the cessation of the offending drug, but chronic hepatic insufficiency is not infrequent.^30–38^

This study helps us understand the predisposing factors, among patients from a wide range of backgrounds and genetic pools in Qatar. It gives us an idea about the incidence and potential concomitant risk factors responsible for ceftriaxone-induced DILI and guides us to improve further the quality of care. In the present study, 19.71% of the patients who received ceftriaxone experienced some liver injuries. This observed incidence is significantly higher than that in the literature. The researchers did not define the type or severity of infectious diseases that were treated with ceftriaxone, and the severity of comorbidities of patients also varied. These factors might have influenced our observations. Although a propensity score presumably helps reduce selection bias, it is difficult and almost impossible to obliterate all confounders.

All common factors suspected to be responsible for hepatitis and deranged liver function tests, such as common viral hepatitis, autoimmune disease, cholelithiasis, and endocrine diseases such as hypo- and hyperthyroidism, were excluded in all patients by blood investigations. In addition, the absence of history of blood transfusion, recent tooth extraction or tattoo, past surgery, direct contact with a patient known to have jaundice, hepatitis, traveling, or use of any concomitant medications, including herbal remedies and vitamins, led us to consider ceftriaxone as the responsible agent.

However, this study has several limitations. First, given the retrospective design, the frequency and timing of blood investigations during and after ceftriaxone treatment were not constant among all eligible patients. Understandably so, data from patients with limited blood investigations might lead to over- or underestimation of the results. Second, the incidence of newly formed biliary sludge or stones after ceftriaxone administration, which are relatively rare but are typical of ceftriaxone adverse effects, was not investigated because not every patient had undertaken an imaging assessment before or after ceftriaxone treatment. Therefore, the association, if any, between hepatic injury and newly construed biliary sludge or calculi is unclear from this study. Third, although this study was conducted in a single center, it consisted of individuals of multinational, multi-racial, and multi-ethnic backgrounds, and we presume that selection bias should not have been an issue. Therefore, considering these issues, prospective multicenter studies are required in the future. Fourth, there were a rather low number of patients who received higher doses of ceftriaxone in our study; hence, the dose relationship was not clearly established or analyzed. Fifth, the researchers understand that being a culturally sensitive issue, many patients might have not been true to their admission of alcohol intake, and this could lead to a potential bias in our study. Sixth, the definition of liver injury is another crucial limitation. For a detailed assessment of DILI, in addition to abnormalities in biochemical parameters, assessments of the pattern, severity, and cause of liver injury are required,^8,17^ and DILI should be diagnosed by exclusion. This review intends to generate veritable data, a first of its kind among the populations in Qatar, and thus embraces a simple screening tool for liver injury by ratifying only biochemical criterion. The researchers further aim to analyze a bigger pool of patients extensively by simultaneously evaluating patterns of liver injury by ceftriaxone.

## Conclusion

DILI remains a challenge to physicians globally treating adult population. Diagnosis by exclusion involves a painstaking process of evaluation along with a high clinical suspicion to identify any drug as the cause of the liver injury. However, the early identification of the clinical and biochemical patterns of hepatotoxicity helps in the prognostication of each patient. With major institutions across the globe now encouraging the expansion of drug injury registries, we hope our understanding of DILI epidemiology, various mechanisms of hepatic injury, and causality will continue to improve. This study demonstrates that treatment of common medical infections with ceftriaxone along with other medications, which also undergo primary hepatic metabolism, was associated with a significantly higher incidence of liver injury among populations in Qatar than in western literature. Furthermore, the incidence was too high to ignore in clinical practice. Over the next decade, DILI research is expected to yield important results that will be translated into clinical practice. Several DILI registries are already mature and expanding worldwide. They will serve as rich reservoirs for clinical and translational research. Until then, DILI remains a problem for clinicians. Its iatrogenic nature and possibility of a severe or deadly outcome can be unsettling for both clinicians and patients. Clinicians worldwide should carefully observe for signs of liver injury during and after the administration of ceftriaxone.

### Acknowledgments

The authors would like to thank the nurses, clerks, laboratory technicians, pharmacists, and resident doctors of our hospital for their contributions to this study. The authors would also like to thank Aaranya Dev Barman for his coding skills, algorithms, and data analysis for this study.

### Author contributions

Muhammad Sharif and Manish Barman conceptualized and designed the study, performed the research, analyzed the data, and wrote the paper. Bassem Al Hariri, Abdul Rahman Mustafa, Naseem Ambra, Mohamedali Gaafar, Memon Noor Illahi, S Hamuda, Israa Amjed, and Ahmad Eid Nazzal Alharafsheh collected and reviewed the data, contributed to the statistical analyses, and critically reviewed the final draft.

Compliance with ethical standards

### Ethical approval

This study was conducted with the approval of the medical research center at Hamad Medical Corporation (Approval no. MRC-01-20-1071).

The ethics committee waived the need for the written informed consent for using participant's sample and analyzing clinical case records because of preserved anonymity.

### Conflict of interest

The authors declare that they have no conflict of interest.

### Funding

No funding was received for this study.

## Figures and Tables

**Table 1:** Drug-induced Liver Injury

Type	Biochemical profile	Prognosis

Hepatocellular	Alanine aminotransferase>2 ULNSerum ALT/serum alkaline phosphatase ≥ 5*	Severe prognosis

Cholestatic	Alkaline phosphatase ≥ 2ULNSerum alanine aminotransferase/serum alkaline phosphatase ≤ 2*	More prone to chronic disease

Mixed	Alanine aminotransferase>2 ULNSerum ALT/serum alkaline phosphatase between 2 and 5^*^	More prone to chronic disease


^*^Values in ratios are expressed as ULN multiples. ULN = upper limit of normal

Biochemical criterion: ALT (ULN = 55 IU/L), AST (ULN = 40 IU/L), ALP (ULN = 120 IU/L), and total bilirubin (TB) ≤ 2.4 mg/dL

**Table 2:** Clinical characteristics of all patients in the multivariate analysis of factors associated with liver injury.

Characteristics		Liver injury	*P* value

Age range	18-85 years		(*p* = 0.98)

< 40 years n (%)	349	67	

41–49 years n (%)	145	29	

>50 years n (%)	140	29	

Male n, (%)	634 (100%)	125	

Female n (%)	XX	XX	

BMI	-	-	(*p* = 0.91)

< 20	70	13	

21–25	323	68	

26–29	158	28	

30–34	62	11	

>35	21	4	

Comorbidities			

Diabetes n (%)	60	17	(*p* = 0.07)

Hypertension n (%)	18	3	(*p* = 0.97)

Others n (%)	75	12	

Site of Infection			(*p* = 0.48)

Pulmonary	422	86	

GI tract	161	33	

Renal	20	3	

Others	31	3	

Concomitant drug			

Azithromycin	195	55	(*p* < 0.01)

Paracetamol	385	85	(*p* < 0.01)

+/- others	439	70	(*p* = 0.01)

Metformin	35	7	(*p* = 0.58)

Calcium channel blocker	12	1	(*p* = 0.27)

Beta blocker	7	1	(*p* = 0.71)

ACEI/ARB	9	1	(*p* = 0.51)

NSAIDs	15	5	(*p* = 0.19)

Alcohol use	66	20	(*p* = 0.53)

Smoking	172	45	(*p* = 0.94)

Outcome			

Liver injury incidence n (%)		125 (19.71%)	


**Table 3:** ANTIBIOGRAM Empiric antibiotics of choice for common clinical infections.

Site of infection	Common causative organism	Empiric antibiotic treatment	Duration

Skin or soft tissue	Uncomplicated cellulitis Strep groups A, B, or C *Staph aureus*/complicated cellulitis	IV: cefazolin/ceftriaxone PO: cephalexin IV: amp/sulbactam +/- vancomycin or piperacillin/tazobactam +/- vancomycin	5–7 days 10–14 days

Bone and Joint	Osteomyelitis, acute *Staphylococcus aureus* (hematogenous) septic arthritis *S. aureus, Streptococcus* spp., gram-negative rods, *Neisseria meningitidis*	Vancomycin +/- ceftriaxone	7–21 days

CNS	Bacterial meningitis, community acquired *S. pneumoniae, N. meningitidis*	High-dose ceftriaxone (2 g q12 h) plus vancomycin	7–14 days

Pneumonia	Community acquired *S. pneumoniae, Mycoplasma, Haemophilus influenzae, Legionella, Moraxella catarrhalis* Less common: *S. aureus,* virus, gram-negative rods ICU patients with CAP	Non-ICU patients: ceftriaxone (1 g q24 h) plus azithromycin (500 mg daily) or levofloxacin alone (750 mg daily) Azithromycin plus ceftriaxone or piperacillin/tazobactam plus (levofloxacin or ciprofloxacin) +/- (vancomycin* or linezolid)	7–14 days

Genito-urinary infection	Cystitis: *E. coli, Staph saprophyticus* Uncomplicated pyelonephritis: *E. coli, Proteus*, other gram-negative rods Complicated pyelonephritis, resistant gram-negative rods, enterococci	PO: sulfamethoxazole/trimethoprim (bid) or cephalexin 500 mg q12 h PO: ciprofloxacin 500 mg bid IV/IM: ceftriaxone 1 g q24 h Ceftriaxone 1 g q24 h or piperacillin/tazobactam	5–14 days

Abdominal	Cholangitis, diverticulitis, bowel perforation, enteric GNR (*Klebsiella, E. coli, Proteus*) +/- Enterococci, anaerobes	Piperacillin/tazobactam alone or ceftriaxone plus metronidazole	5–10 days

